# Trans-Coronary Sinus Intra-Septal Radiofrequency Ablation (TIRA) for Hypertrophic Obstructive Cardiomyopathy: First-in-Human Results

**DOI:** 10.3390/biomedicines12122762

**Published:** 2024-12-04

**Authors:** Ji-Soo Oh, Jae-Young Seo, Cheol-Min Lee, Su-Jin Jung, June-Hong Kim, Min-Ku Chon

**Affiliations:** 1 Department of Internal Medicine, Cardiovascular Center, Ulsan Hospital, Ulsan 44686, Republic of Korea; oj033039@gmail.com; 2 Department of Internal Medicine, School of Medicine, Pusan National University, Busan 49241, Republic of Korea; shope2024@pusan.ac.kr; 3 Department of R&D Center, Tau Medical Inc., Busan 50612, Republic of Korea; 4 Department of Internal Medicine, Pusan National University School of Medicine and Cardiology, Cardiovascular Center and Research Institute for Convergence of Biomedical Science and Technology, Pusan National University Yangsan Hospital, Yangsan 50612, Republic of Korea; junehongk@gmail.com

**Keywords:** hypertrophic obstructive cardiomyopathy (HOCM), left ventricular outflow tract (LVOT) obstruction, TIRA-HOCM device, coronary venous system access, first in-human clinical trial

## Abstract

Background: Current treatments for hypertrophic obstructive cardiomyopathy (HOCM), including medication, surgery, and alcohol septal ablation (ASA), have limitations in terms of efficacy and safety. To address these challenges, we developed the trans-coronary intra-septal radiofrequency ablation (TIRA) device. Methods: This first-in-human trial was conducted to assess the efficacy and safety of the TIRA device. Moreover, evaluations were conducted before the procedure and at 3, 6, and 12 months post-procedure using computed tomography, magnetic resonance imaging, echocardiography, and the 6 min walk distance (6MWD) test. Results: Four patients were enrolled, and follow-up imaging at 3, 6, and 12 months showed a reduction in the interventricular septal (IVS) thickness (baseline mean: 22.6 mm; 12-month mean: 18.9 mm) and a decrease in the LVOT pressure gradient at 12 months (resting baseline mean: 84.64 mmHg; resting 12-month mean: 43.56 mmHg; Valsalva baseline mean: 129.96 mmHg; Valsalva 12-month mean: 108.16 mmHg). However, reductions in the IVS thickness on echocardiography and improvements in 6MWD were observed in only two patients. Conclusions: No significant adverse events, such as arrhythmias or vascular injuries, were reported. These findings suggest that the TIRA device may be a safe and effective option for treating HOCM. However, further studies are required to confirm these results.

## 1. Introduction

Hypertrophic obstructive cardiomyopathy (HOCM) is a genetic cardiac disorder characterized by an abnormal thickening of the interventricular septum that leads to left ventricular outflow tract (LVOT) obstruction and restricted blood flow from the heart. This dynamic obstruction results in symptoms such as dyspnea, angina, syncope, and, in severe cases, sudden cardiac death. HOCM is relatively rare, affecting approximately 0.2% of the population, and is classified as a genetic disorder with considerable clinical variability, based on the degree of septal hypertrophy and LVOT obstruction [1,2,3,4]. The condition is most often associated with mutations in genes encoding sarcomeric proteins that cause disorganized myofibrils and abnormal myocardial cell growth [5]. This genetic heterogeneity contributes to variable disease progression and symptom severity, necessitating tailored treatment strategies. The primary goal of HOCM management is to reduce LVOT obstruction and alleviate symptoms. Pharmacological treatments, including beta-blockers, calcium channel blockers, and disopyramide, are typically the first line of therapy and help reduce the contractility of the heart and the severity of the obstruction [6]. However, despite the availability of these drugs, many patients, particularly those with significant septal hypertrophy, experience insufficient symptom relief [7]. In recent years, new pharmacological agents, such as Mavacamten, which is a selective cardiac myosin inhibitor, have been developed to target the underlying pathophysiology of HOCM by reducing hypercontractility [8]. Although Mavacamten has shown promise in lowering LVOT gradients and improving symptoms, it is not universally effective, and its long-term efficacy and safety in a broader population remain to be fully established [9]. For patients who do not respond adequately to medical therapy, more invasive options are considered. Surgical septal myectomy has long been regarded as the gold standard treatment for severe HOCM. This procedure involves the surgical removal of a portion of the thickened septum to reduce the obstruction [10]. Although highly effective, septal myectomy is an open-heart surgery with inherent risks, including prolonged recovery times, bleeding, and potential damage to surrounding structures. As a result, many patients and clinicians seek less invasive alternatives [11]. In response to the limitations of pharmacological and surgical interventions, several minimally invasive procedures have been developed. Alcohol septal ablation (ASA) is one such procedure that has gained traction. ASA involves the injection of ethanol into a septal branch of the coronary artery, inducing localized necrosis and the thinning of the septum [12]. However, ASA carries significant risks, such as arrhythmias, ventricular septal defects, and an incomplete reduction in septal thickness, especially in patients with complex coronary anatomy. Furthermore, the efficacy of the procedure is limited by the challenge of precisely controlling the extent of myocardial necrosis, which can lead to inconsistent clinical outcomes [13]. Another promising minimally invasive approach is percutaneous intramyocardial septal radiofrequency ablation (PIMSRA). This procedure uses a needle inserted through the chest wall to deliver radiofrequency energy directly to the hypertrophic septum and thereby reduce septal thickness and alleviate LVOT obstruction [14]. Although PIMSRA avoids the need for open-heart surgery, it presents its own set of challenges and risks. Chest wall penetration increases the potential for infection, bleeding, and trauma to adjacent structures, including the lungs and coronary arteries. Additionally, accurately targeting the hypertrophic tissue can be difficult, which could lead to suboptimal outcomes and incomplete symptom relief in some cases [15]. To overcome the limitations of these existing therapies, we developed a novel transcatheter-based device, namely, the transcatheter intramyocardial radiofrequency ablation (TIRA). This device was designed to offer a safer and more controlled approach to septal reduction by utilizing the coronary venous system for access rather than puncturing the chest wall [16]. Unlike PIMSRA, the TIRA-HOCM device employs a trans-coronary sinus approach that allows for the delivery of radiofrequency energy directly to the hypertrophic septal tissue through the coronary venous system. By using the venous system, the procedure avoids many of the risks associated with arterial interventions and chest wall penetration, such as infection and bleeding. Moreover, the trans-coronary sinus route provides a stable and consistent anatomical pathway that reduces the risk of mistargeting and enhances procedural accuracy [17]. In preclinical studies, TIRA-HOCM was evaluated in a total of 11 pigs (7 survival, 4 non-survival) to assess its efficacy and safety. These experiments demonstrated reductions in septal thickness and improvements in LVOT hemodynamics. No major complications were observed. The device effectively provided targeted ablation while minimizing collateral damage to surrounding tissues, including coronary vessels and conduction pathways. Encouraged by these preclinical findings, the device was further refined to enhance flexibility and better accommodate patient-specific anatomy to ensure its suitability for use in future human trials. The refinement process involved optimizing the flexibility of the catheter and ablation energy settings to maximize safety and efficacy during human application. With these advancements, we initiated the first in-human clinical trial to assess the safety and efficacy of the TIRA-HOCM device in patients with symptomatic HOCM who were refractory to conventional medical therapy. This study aimed to evaluate key efficacy endpoints, such as reductions in septal thickness, improvements in LVOT obstruction, and the overall safety of the procedure. The development of TIRA-HOCM presents a novel, minimally invasive treatment option that may offer significant advantages over existing therapies, potentially reduce procedural risks, and provide more consistent and durable clinical outcomes.

## 2. Methods

### 2.1. TIRA-HOCM Device

**Figure 1:**
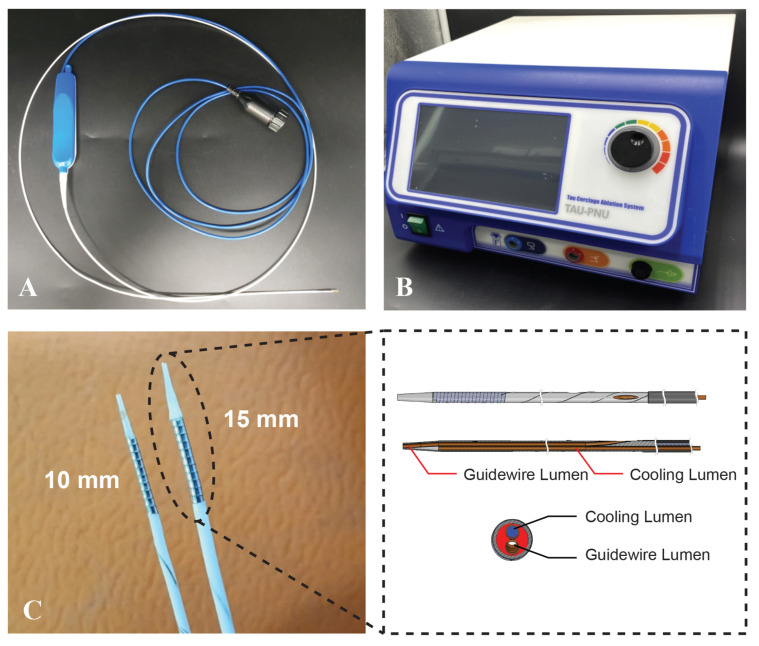
Overview of the TIRA device and associated components. (**A**) TIRA device, (**B**) the radio frequency generator connected to the TIRA device, which delivers controlled energy during the procedure. (**C**) A detailed view of the TIRA device, highlighting key components such as the coil electrode and cooling lumen.

The TIRA ablation system (Figure 1) was designed as a catheter-based ablation device for targeted myocardial tissue reduction. This investigational medical device is a monopolar ablation catheter with a diameter of 4 Fr and offers coil electrodes in two lengths, that is, 10 mm and 15 mm, to specifically target the hypertrophic ventricular septum. The catheter facilitates percutaneous and transvascular access to the coronary sinus via the right internal jugular vein, which allows for the precise placement within the septal vein to reach the interventricular septum. A cooling lumen within the catheter enables the infusion of saline to cool the coil electrode, which prevents carbonization during the ablation process. The coil electrode, which is located at the distal tip of the device, ensures the direct delivery of RF energy into the myocardial tissue to achieve localized tissue ablation. The distal shaft of the catheter contains wires that transmit RF energy from the power source to the coil electrode along with thermocouples to continuously monitor the temperature during the procedure. The ablation system is powered by a 480 kHz radiofrequency (RF) generator (Myogen CAS-20, RF Medical, Ltd., Seoul, Republic of Korea). This generator operates in a temperature-controlled mode and automatically pauses the ablation if the impedance exceeds 250 ohms, thus ensuring safety and precision. To facilitate ablation, four ground pads are placed in a parallel (2 × 2) configuration on the waist of the patient. This configuration ensures efficient energy delivery and stable ablation performance throughout the procedure.

### 2.2. Patients and Study Design

This open-label, single-arm, prospective, first-in-human feasibility study was approved by the Korea Food and Drug Administration and the Institutional Review Board of Pusan National University Yangsan Hospital (NCT04770142). This study enrolled patients who provided written informed consent and were carefully selected by the institutional multidisciplinary heart team. Eligible participants were required to have persistent New York Heart Association (NYHA) functional class II or higher heart failure symptoms, despite more than three months of guideline-directed optimal medical therapy [18], along with a left ventricular outflow tract (LVOT) gradient of ≥30 mmHg at rest or ≥50 mmHg during physiological stress, dobutamine infusion, or the Valsalva maneuver, with the gradient caused by the systolic anterior motion (SAM) of the mitral valve leading to proximal mitral valve–septal contact. Patients were excluded if they had a ventricular septal wall thickness of less than 15 mm in the target ablation area or left ventricular ejection fraction (LVEF) of ≤40%. Additionally, patients were excluded if computed tomography (CT) or venography revealed a coronary sinus or septal vein anatomy that was incompatible with the procedure. Further exclusions applied to individuals with a conduction disturbance, such as a left or right bundle branch block (LBBB or RBBB), an advanced AV block without a permanent pacemaker, a recent percutaneous coronary intervention (PCI) or coronary artery bypass grafting (CABG) near the coronary sinus or adjacent veins within the previous three months, severe pulmonary hypertension (pulmonary arterial pressure of >70 mmHg), cardiogenic shock, or an expected life expectancy of less than 12 months. Pregnant or breastfeeding women, women planning to conceive, or those not using medically approved contraception were also excluded, as were individuals who had participated in another clinical trial within 30 days prior to screening, or those who were deemed inappropriate by the investigator, owing to clinically significant conditions. The primary efficacy endpoints were the morphological changes in the ventricular septum and the reduction in LVOT obstruction, with septal thickness assessed via CT and MRI at baseline and at 1, 3, 6, and 12 months post-procedure. Moreover, echocardiography with Doppler measurements was used to monitor LVOT gradients at each follow-up to confirm hemodynamic improvements. If a participant had a pacemaker implanted, MRI imaging could not be performed, and such imaging assessments were excluded for that individual. Procedural safety was evaluated by monitoring for adverse events, such as arrhythmia, myocardial infarction, or vascular injury during and after the procedure. Of the four participants, three were female (75%) and one was male (25%), with a mean age of 69.5 ± 13.4 years. All patients were diagnosed with dyslipidemia. Additionally, three (75%) had hypertension, one (25%) had chronic atrial fibrillation, one (25%) had angina pectoris, and one (25%) had a pacemaker implantation. This report analyzes data from the four patients who completed both the TIRA procedure and the entire 12-month follow-up (Table 1).

### 2.3. Procedure

**Figure 2:**
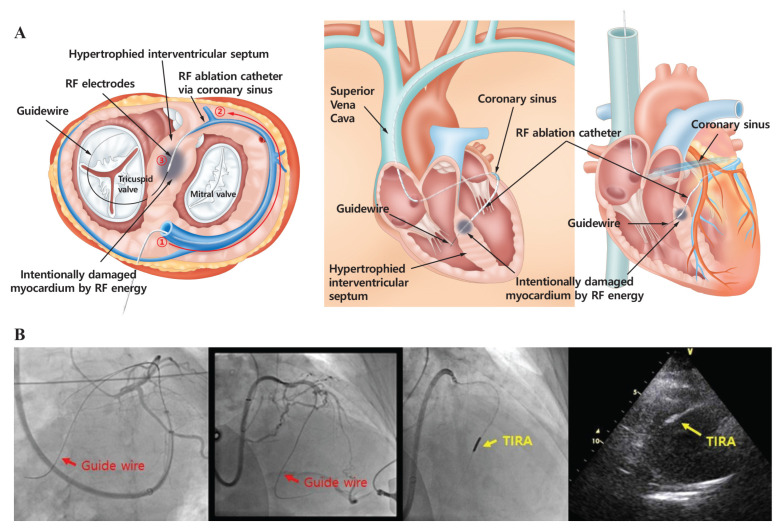
Route of the TIRA Catheter from the jugular vein to the coronary sinus and septal perforator veins. (**A**) An illustration of the TIRA procedure, showing the catheter route starting from the jugular vein (①), passing through the coronary sinus (②), and reaching the target septal veins (③). (**B**) Confirmation of the catheter’s movement during the TIRA procedure using angiography.

**Figure 3:**
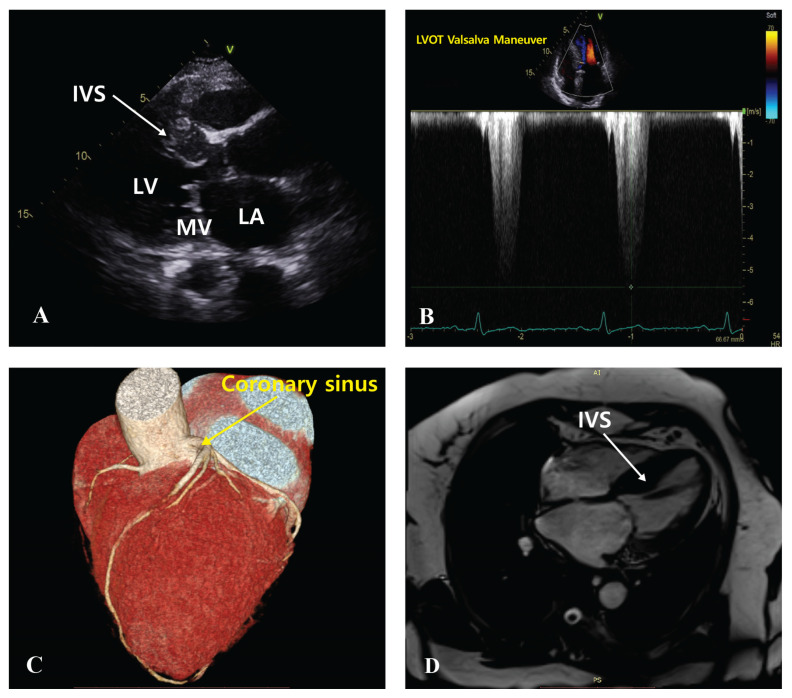
Imaging of IVS and LVOT using echocardiogram, CT, and MRI (pre-procedure). (**A**) Echocardiographic image showing the interventricular septum (IVS) with white arrows. (**B**) Echocardiographic image showing the measurement of the LVOT gradient during a Valsalva maneuver. (**C**) A 3D snapshot from CT imaging showing the anatomical structure of the coronary sinus. (**D**) MRI image showing the baseline IVS.

Prior to the procedure, coronary CT angiography with three-dimensional (3D) reconstruction was used to assess the cardiac anatomy of each patient and identify suitable septal perforator veins. This thorough pre-procedural planning enabled the precise delivery of the TIRA ablation catheter to the target site within the interventricular septum (Figure 2). All procedures were conducted under conscious sedation with real-time fluoroscopy and transthoracic echocardiography (TTE) monitoring (Figure 3). The jugular vein served as the primary access route for the ablation catheter, whereas the femoral artery and vein were used for coronary angiography and temporary pacemaker placement to reduce atrioventricular (AV) block risk. A 6 Fr double-curve guiding catheter (Renal Curve Adult, Cordis, Miami, FL, USA) was introduced into the coronary sinus (CS) to access the septal perforator veins. Using an 8-French balloon-tipped guiding catheter (Cello catheter, Medtronic, Dublin, Ireland), the coronary sinus was engaged. A contrast venogram identified the target septal vein. A 0.014-inch coronary guidewire (Asahi Sion, Conquest, or Astato wire, Asahi Intecc, Aichi, Japan) was then advanced into the target vein with a dual-lumen microcatheter (Crusade catheter, Kaneka Corporation, Tokyo, Japan). The TIRA ablation catheter was subsequently guided along the wire to the interventricular septum. In the ablation process, the device was first positioned at the innermost point of the target site. Ablation was initiated at 60 °C and gradually increased to a maximum of 80 °C in 5 °C increments, with each cycle lasting 2–3 min to create effective lesions. Irrigation was performed using saline at a flow rate of 2 cc/min to prevent electrode overheating and carbonization during the procedure. No steam pops were observed, indicating stable energy delivery and effective temperature control. Then, the device was pulled back slightly along the guidewire for each subsequent ablation. This process was repeated three times. The procedure was performed with continuous TTE and fluoroscopic monitoring to ensure catheter stability and optimal positioning. Upon completion, coronary angiography was repeated to confirm the integrity of the coronary sinus. Among the four patients, no arrhythmias or AV node conduction blocks were observed post-procedure, and there were no significant complications. The average procedure time was 205.0 ± 17.3 min.

## 3. Results

**Figure 4:**
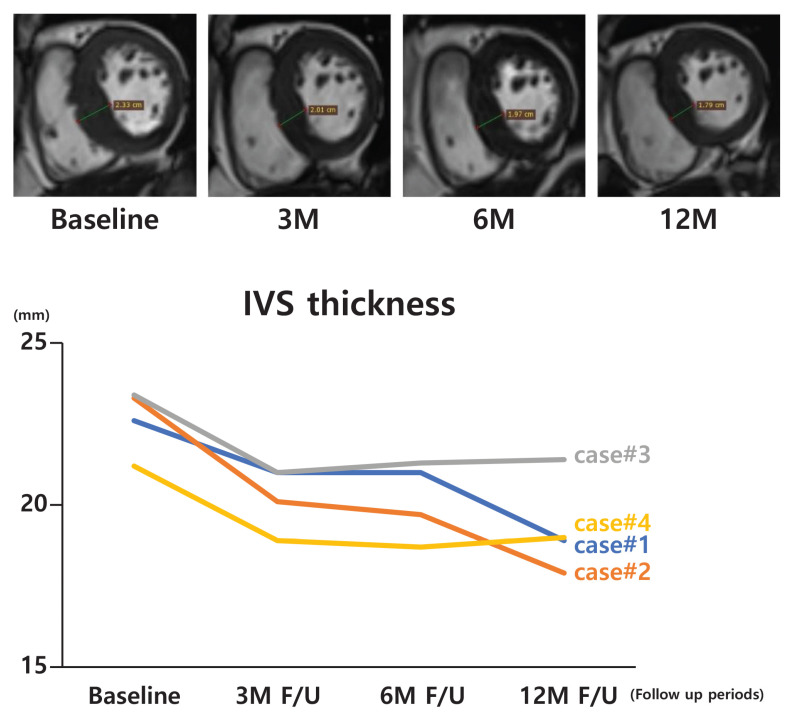
CT or MRI measurements of interventricular septal thickness taken during the follow-up period demonstrated a reduction in septal thickness over time.

The primary efficacy endpoints involved assessing morphological changes in the ventricular septum through computed tomography (CT) and magnetic resonance imaging (MRI) to observe variations in septal thickness at the targeted site. Imaging was conducted at intervals of 3 d, 1 m, 3 m, 6 m, and 12 m following the TIRA-HOCM procedure to monitor septal remodeling over time. RadiAnt DICOM Viewer 2021.2 (64-bit) was utilized to analyze both the CT and MRI data. For Case 1, in which the patient had a pacemaker and was unable to undergo MRI, septal thickness was evaluated via CT in the four-chamber view, showing a reduction in IVS thickness and suggesting the effective remodeling of the hypertrophic tissue. For the remaining three patients, MRI was used as the primary imaging modality, owing to its superior soft-tissue resolution. Measurements were focused on the thickest region of the IVS using the cine-segmented view. All four patients demonstrated a steady reduction in IVS thickness throughout the follow-up period, indicating the potential of the TIRA procedure to alleviate LVOT obstructions. By the 3-month follow-up, a decrease in IVS size was observed in all patients. Of these, two cases showed further reductions up to the 12-month follow-up point, suggesting continued remodeling, whereas the other two cases remained stable beyond the 3-month follow-up point. Defect sizes were monitored over time for the three patients with MRI follow-up data, and a gradual decrease was observed. These findings suggest that the ablated area may undergo progressive shrinkage over time and contribute to reduced septal thickness. This structural improvement, which is consistent across all cases, aligns with improvements in cardiac function and symptom relief, thus underscoring the potential utility of the TIRA procedure for patients with obstructive HOCM (Figure 4) (Table 2).

**Figure 5:**
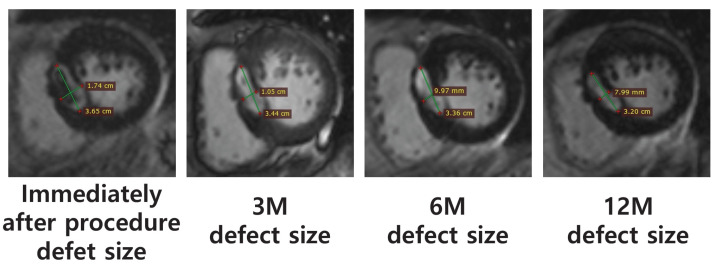
Changes in defect size over the follow-up period, based on MRI measurements.

Among the four patients, MRI follow-ups were conducted for the three patients who were eligible for MRI imaging. The defect size following the TIRA-HOCM procedure was measured for these three patients at multiple time points, including immediately post-procedure, 3 months (3 M), 6 months (6 M), and 12 months (12 M), to monitor the progress of septal remodeling over time. These measurements were performed using the cine-segmented view of the MRI, which is a dynamic imaging sequence that provides high-resolution visualizations of myocardial structures. To ensure consistency, the defect size for each patient was assessed on the same cine-segmented view across all follow-up sessions, thus minimizing measurement variability and enhancing data reliability. The defect size was quantified by recording both the long and short axis dimensions. The long axis was defined as the maximum length of the defect and captured the most extended region of the ablated tissue, whereas the short axis was the shortest measurement across the same plane. This approach ensured that the full extent of morphological changes in the defect could be accurately tracked over time, thus providing a detailed understanding of how the ablated area responded to the intervention. All three patients who underwent MRI showed a progressive reduction in defect size throughout the follow-up period. This reduction reflects the shrinkage and recovery of tissue following IVS ablation, thus indicating the successful remodeling of the hypertrophic tissue. These structural improvements contributed to the alleviation of LVOT obstruction and demonstrated the effectiveness of TIRA in improving both cardiac function and symptom relief (Figure 5) (Table 3).

The echocardiographic data were reviewed and analyzed by an experienced echocardiography specialist. Throughout the follow-up period, IVS thickness was measured at regular intervals to track changes in the septal morphology. Additionally, the LVOT gradient was measured under resting conditions and during the Valsalva maneuver. The LVOT diameter was also assessed to identify any structural changes in the outflow tract. All measurements were consistently performed at each follow-up to ensure reliable data collection. In terms of septal thickness, the second patient exhibited a notable reduction in septal thickness on echocardiography, whereas the other patients showed minimal changes in septal thickness on echocardiography, compared with the more pronounced reductions observed on CT/MRI. Nevertheless, a reduction in the LVOT pressure gradient was observed in most patients at the 12-month follow-up, indicating improved outflow dynamics and supporting the efficacy of TIRA in alleviating LVOT obstruction. Similarly, the LVOT pressure gradient during the Valsalva maneuver demonstrated an overall decrease in all patients. However, the LVOT diameter remained largely unchanged throughout the follow-up period (Table 4).

In this TIRA-HOCM clinical trial, four patients were treated with an investigational medical device, and functional capacity was assessed using the 6 min walk distance (6MWD) test. Case 1 was unable to complete the 6MWD at the 3-month follow-up owing to functional decline from an unrelated cause and temporary medication discontinuation. However, Case 1 completed the 6MWD test at the 6-month and 12-month follow-ups, with recovery observed at the 12-month mark after pharmacological intervention. Cases 2 and 3 demonstrated improvements in their 6MWD results, reflecting enhanced post-procedure functional status, whereas Case 4 showed minimal change across the follow-up period. Overall, an increase in exercise tolerance was noted in most patients, as reflected by improvements in the 6MWD. Additionally, there were no significant changes in other laboratory parameters, including renal function, liver function, and other routine blood tests, in any of the patients throughout the study period, suggesting that the device was well tolerated and did not adversely affect these organ systems (Table 5).

## 4. Discussion

Hypertrophic obstructive cardiomyopathy (HOCM) is managed using surgical, medical, and invasive treatments, each with specific benefits and risks. Surgical treatment, such as septal myectomy, involves removing the thickened portion of the septum to relieve LVOT obstruction and is considered the gold standard for severe cases. However, it is an open-heart procedure with risks including infection, bleeding, and arrhythmia. Medical treatments include beta blockers, calcium channel blockers, or disopyramide, which help reduce heart rate and LVOT obstruction to alleviate symptoms. However, patients with advanced hypertrophy may not always respond adequately to medications alone, thus necessitating additional interventions. Invasive treatments are less invasive alternatives to surgery. Alcohol septal ablation (ASA) induces myocardial infarction by injecting ethanol into the septal perforator arteries to reduce the septal thickness. Although ASA is effective, it has limitations, such as incomplete septal reduction, residual LVOT gradients, and potential complications, such as arrhythmia or heart block, which often require pacemaker implantation. Another minimally invasive option is percutaneous ablation, which targets hypertrophic tissues using energy. However, precise targeting can be challenging, especially in patients with a complex septal anatomy, which sometimes results in incomplete symptom relief. Bipolar ablation has the potential to significantly enhance ablation efficacy compared to monopolar ablation. While the current TIRA-HOCM device is limited to a monopolar system due to technical constraints, implementing a bipolar system represents a critical area for future development. Future studies should assess the feasibility of this approach and its impact on procedural outcomes. To overcome these challenges, the TIRA-HOCM procedure has been developed as a safer and more precise approach. This transcatheter-based treatment delivers radiofrequency ablation (RFA) energy through the coronary venous system to ensure the targeted ablation of the hypertrophic septum. During and after ablation, the reduction in tissue volume leads to measurable myocardial shrinkage, which is reflected by decreased interventricular septal (IVS) thickness. This controlled tissue reduction demonstrated successful septal remodeling, thus contributing to better hemodynamic performance and symptom relief [19]. The cooling system of the current TIRA-HOCM device was designed with an irrigation rate of 2 cc/min based on preclinical studies to balance safety and efficacy. However, we acknowledge that increasing the irrigation rate could enhance lesion size, provided the vein size permits this adjustment. Future iterations of the device should explore this modification to further optimize ablation outcomes, and clinical trials will be needed to evaluate its effectiveness. Moreover, CT and MRI confirmed significant IVS reduction, thus validating the structural changes achieved through this procedure. In contrast, echocardiographic measurements of the IVS thickness and LVOT diameter revealed minimal changes over 12 months. This discrepancy highlights the limitations of echocardiography, including challenges with real-time measurements and patient movement during examinations. Although echocardiography can detect meaningful reductions in patients with significant IVS thinning on MRI or CT, minor reductions of 2–3 mm are difficult to accurately measure using echocardiography [20]. The TIRA-HOCM procedure also achieved reductions in LVOT gradients under both resting conditions and the Valsalva maneuver, although some variability in outcomes was observed, likely because of the small sample size and other comorbidities. In Case 1, the LVOT gradient significantly increased at the 6-month follow-up, likely because the patient independently discontinued all medications. Upon resuming the prescribed medications, the LVOT gradient improved. In terms of exercise capacity, most patients showed improvements in their 6 min walk distance (6MWD) test results, reflecting enhanced functional outcomes. Notably, patients with a more significant IVS reduction tended to show greater increases in the 6MWD, further supporting the correlation between structural improvement and functional capacity. However, Cases 1 and 4 experienced limited progress in the 6MWD, possibly owing to age-related factors and joint conditions, such as arthritis, which may have impaired their mobility. Overall, the TIRA-HOCM procedure demonstrated a favorable safety profile with no significant adverse events, such as arrhythmias or vascular injuries. However, potential risks associated with the TIRA procedure should be acknowledged, including venous injury, thrombus formation, and arrhythmias such as atrioventricular block (AVB) or scar-related ventricular tachycardia (VT). These risks are well-documented in other septal reduction therapies, and their absence in this study may reflect the limited sample size (*n* = 4) rather than an elimination of these possibilities. To mitigate these risks, the study incorporated real-time imaging modalities such as echocardiography and fluoroscopy to guide catheter placement, ensuring precise targeting and minimizing vascular trauma. Additionally, anticoagulant therapy was administered to reduce the risk of thrombus formation, and continuous ECG monitoring was employed to detect any arrhythmic events during and after the procedure. Future studies with larger sample sizes and longer follow-up periods will be essential to further evaluate the safety profile of the TIRA procedure and monitor for potential late-onset complications. In this study, we compared the results of the TIRA-HOCM procedure with those reported for alcohol septal ablation (ASA) and Mavacamten therapy. While the small sample size (*n* = 4) in our study limits the ability to draw definitive conclusions, we observed that the reduction in LVOT gradients achieved with TIRA was comparable to the outcomes reported in studies on ASA and Mavacamten. ASA achieves gradient reduction through targeted myocardial infarction in the septum but carries risks such as arrhythmias and incomplete septal reduction. Mavacamten, as a pharmacological approach, effectively reduces LVOT gradients by modulating myocardial contractility, offering an alternative for patients who may not be suitable candidates for invasive procedures. In comparison, TIRA represents a minimally invasive option that directly targets septal thickness [21,22]. However, the variability in LVOT gradient reduction across patients suggests the need for further investigation. Therefore, large-scale studies with longer follow-up periods are essential to confirm the efficacy of the procedure and refine the protocol for wider clinical use. Another promising application of the TIRA-HOCM device is for the treatment of septal ventricular tachycardia (VT). The venous-based access method utilized by the device provides a safe and effective route for targeting deep septal lesions, which are often challenging to reach with conventional approaches. Future clinical trials should explore the efficacy and safety of the TIRA-HOCM device in this context, as it may offer an innovative solution for septal VT ablation. With continued research and optimization, TIRA-HOCM can become a reliable, safe, and effective treatment for obstructive hypertrophic cardiomyopathy.

## 5. Conclusions

The TIRA-HOCM procedure demonstrated a strong safety profile and promising reduction in septal thickness and LVOT gradients, positioning it as a viable, minimally invasive alternative to current treatments. However, further studies with larger patient populations and extended follow-up periods are required to confirm its long-term efficacy and broader applicability.

## Figures and Tables

**Table 1:** Baseline characteristics (*n* = 4).

Case	Sex/Age (yrs)	NYHA	Medical History					
			Hypertension	Dyslipidemia	A. Fib	Angina Pectoris	Pacemaker	Imaging Guidance
1	F/79	2	−	+	+	−	+	CT TTE
2	F/66	2	+	+	−	−	−	CT MRI TTE
3	M/52	2	+	+	−	−	−	CT MRI TTE
4	F/81	2	+	+	−	+	−	CT MRI TTE

**Table 2:** Interventricular septal thickness measurement (*n* = 4).

Case	Computed Tomography or MRI Measurement Data (Interventricular Septal Thickness)			
	Baseline	3 M	6 M	12 M
1 *	22.6	21.0	-	18.9
2	23.3	20.1	19.7	17.9
3	23.4	21.0	21.3	21.4
4	21.2	18.9	18.7	19.0

* Case 1 was unable to undergo MRI imaging owing to the implantation of a pacemaker, which is contraindicated for MRI procedures.

**Table 3:** Ablation defect size measurement via MRI.

Case	Ablation Defect Size (MRI, mm)			
	Immediately Following Procedure	3 M	6 M	12 M
1	-	-	-	-
2	36.5 × 17.4	34.4 × 10.5	33.6 × 10.0	32.0 × 8.0
3	25.7 × 18.9	16.6 × 11.4	14.4 × 7.8	13.0 × 7.7
4	12.6 × 12.3	11.3 × 10.5	9.6 × 10.1	9.7 × 8.7

**Table 4:** Echocardiogram measurement (*n* = 4).

Case	Echocardiogram Data			
	IVS Thickness (mm, Diastolic)			
	Baseline	3 M	6 M	12 M
1	17.2	18.4	18.1	17.8
2	23.2	21.2	21.7	20.6
3	23.5	23.4	23.1	23.0
4	21.4	22.5	22.5	22.6
	**LVOT Gradient (mmHg): Resting Maneuver**			
1	84.64	92,16	125.44	43.56
2	100.00	54.76	46.24	38.44
3	40.96	36.00	25.00	25.00
4	112.36	116.64	92.16	100.00
	**LVOT Gradient (mmHg): Valsalva Maneuver**			
1	129.96	134.56	219.04	108.16
2	129.96	116.64	54.76	43.56
3	70.56	70.56	38.44	33.64
4	184.96	148.84	121.00	148.84
	**LVOT Diameter (mm)**			
1	15.2	15.6	16.1	15.6
2	20.5	16.5	17.0	17.2
3	17.5	18.3	17.9	18.0
4	21.9	21.9	21.8	22.1

**Table 5:** Changes in patients 6MWD before and after the procedure during the follow-up period.

Case	Laboratory Data			
	6MWD (m)			
	Baseline	3 M	6 M	12 M
1	315	-	340	303
2	300	405	410	375
3	375	385	410	405
4	316	303	320	320

## Data Availability

Clinical Affiliation: clinicaltrials.gov (accessed on 19 November 2024) Identifier: NCT04770142.

## References

[1] Maron B.J., Gardin J.M., Flack J.M., Gidding S.S., Kurosaki T.T., Bild D.E. (1995). Prevalence of hypertrophic cardiomyopathy in a general population of young adults: Echocardiographic analysis of 4111 subjects in the CARDIA study. Circulation.

[2] Semsarian C., Ingles J., Maron M.S., Maron B.J. (2015). New perspectives on the prevalence of hypertrophic cardiomyopathy. J. Am. Coll. Cardiol..

[3] Lopes L.R., Ho C.Y., Elliott P.M. (2024). Genetics of hypertrophic cardiomyopathy: Established and emerging implications for clinical practice. Eur. Heart J..

[4] Maisch B., Mahrholdt H. (2014). The 2014 ESC guidelines on the diagnosis and management of hypertrophic cardiomyopathy: What is new?. Herz.

[5] Niimura H., Bachinski L.L., Sangwatanaroj S., Watkins H., Chudley A.E., McDonough B., Wakimoto H., Setoguchi M., Yubeta T., Nakada K. (2002). Sarcomere protein gene mutations in hypertrophic cardiomyopathy of the elderly. Circulation.

[6] Sherrid M.V., Shetty A., Winson G., Palacios I., McKenna W.J., Elliott P.M., Rahman S., Wheeler M., Dinkes R., Treat J. (2013). Treatment of obstructive hypertrophic cardiomyopathy symptoms and gradient resistant to first-line therapy with β-blockade or verapamil. Circ. Heart Fail..

[7] Maekawa Y., Akita K., Takanashi S. (2018). Contemporary septal reduction therapy in drug-refractory hypertrophic obstructive cardiomyopathy. Circ. J..

[8] Tower-Rader A., Ramchand J., Nissen S.E., Desai M.Y. (2020). Mavacamten: A novel small molecule modulator of β-cardiac myosin for treatment of hypertrophic cardiomyopathy. Expert Opin. Investig. Drugs.

[9] Savage P., Mahfoud F., Freedman B., O’Donoghue M., Clarke J., Dunne L., Schulman S.P., Selvanayagam J.B., Andrews J., Moses J.W. (2022). Advances in clinical cardiology 2021: A summary of key clinical trials. Adv. Ther..

[10] Brown M.L., Schaff H.V. (2008). Surgical management of obstructive hypertrophic cardiomyopathy: The gold standard. Expert Rev. Cardiovasc. Ther..

[11] Maron B.J., Nishimura R.A. (2014). Surgical septal myectomy versus alcohol septal ablation: Assessing the status of the controversy in 2014. Circulation.

[12] Masry H.E., Breall J.A. (2008). Alcohol septal ablation for hypertrophic obstructive cardiomyopathy. Curr. Cardiol. Rev..

[13] Califf R.M., Abdelmeguid A.E., Kuntz R.E., Popma J.J., Davidson C.J., Cohen E.A., Kleiman N.S., Mahaffey K.W., Topol E.J., Ohman E.M. (1998). Myonecrosis after revascularization procedures. J. Am. Coll. Cardiol..

[14] Liu L., He Y., Chen R., Luo J., Zhou X., Chen Z., Zhao H., Jiang L., Zhuang W., Li J. (2018). Percutaneous intramyocardial septal radiofrequency ablation for hypertrophic obstructive cardiomyopathy. J. Am. Coll. Cardiol..

[15] Wolfram D., Tzankov A., Pülzl P., Piza-Katzer H. (2009). Hypertrophic scars and keloids—A review of their pathophysiology, risk factors, and therapeutic management. Dermatol. Surg..

[16] Shin E.-S., Nair P.B., Kim J.-S., Choe Y.H., Baek S.H., Jeong J.O., Park J.-C., Kang S.-H., Yoon Y.E., Yang D.-H. (2020). Septal reduction using transvenous intramyocardial cerclage radiofrequency ablation: Preclinical feasibility. Basic Transl. Sci..

[17] Park Y.-H., Sun B.J., Lee P.H., Jung S.H., Kim D.H., Song J.K., Lee J.W., Kim J.-J., Na C.Y., Lim S.-H. (2017). Mitral loop cerclage annuloplasty for secondary mitral regurgitation: First human results. JACC Cardiovasc. Interv..

[18] Bennett J.A., Riegel B., Bittner V., Nichols J. (2002). Validity and reliability of the NYHA classes for measuring research outcomes in patients with cardiac disease. Heart Lung.

[19] Barkagan M., Riahi S., Choi S.H., Lazzara R., Liem L.B., Deyell M., Kelen G., Gokce A., Trachtenberg B., Coelho D. (2019). Histopathological characterization of radiofrequency ablation in ventricular scar tissue. JACC Clin. Electrophysiol..

[20] Puntmann V.O., Tzikas S., Kozerke S., Hess M., Bremerich J., Nagel E., Gebker R., Uecker M., Schuster A., Rees C. (2013). Left ventricular chamber dimensions and wall thickness by cardiovascular magnetic resonance: Comparison with transthoracic echocardiography. Eur. Heart J.–Cardiovasc. Imaging.

[21] Oktay V., Arslan S., Gecit M.H., Bulat Z., Gokce M.E. (2024). Short-and Mid-Term Outcomes of Early Alcohol Septal Ablation Therapy for Patients with Mildly Symptomatic Hypertrophic Obstructive Cardiomyopathy: A Tertiary Center Experience. J. Clin. Med..

[22] Desai M.Y., Zain A., Bhatia M., Smedira N., Patel P., Sideris E., Kwan J., Mahajan N., Kellett M., Ray I. (2022). Myosin inhibition in patients with obstructive hypertrophic cardiomyopathy referred for septal reduction therapy. J. Am. Coll. Cardiol..

